# Changes in Volatile Compound Profiles in Cold-Pressed Oils Obtained from Various Seeds during Accelerated Storage

**DOI:** 10.3390/molecules26020285

**Published:** 2021-01-08

**Authors:** Anna Gaca, Eliška Kludská, Jaromír Hradecký, Jana Hajšlová, Henryk H. Jeleń

**Affiliations:** 1 Faculty of Food Science and Nutrition, Poznań University of Life Sciences, Wojska Polskiego 31, 60-624 Poznań, Poland; anna.gaca@up.poznan.pl; 2 Department of Food Analysis and Nutrition, University of Chemistry and Technology, 166 28 Prague 6, Czech Republic; e.humlova@seznam.cz (E.K.); hradecky@fld.czu.cz (J.H.); jana.hajslova@vscht.cz (J.H.); 3 Faculty of Forestry and Wood Sciences, Czech University of Life Sciences, 165 00 Prague, Czech Republic

**Keywords:** cold-pressed oil, GC-HRToFMS, multivariate analysis, volatile compounds

## Abstract

Cold-pressed oils are highly valuable sources of unsaturated fatty acids which are prone to oxidation processes, resulting in the formation of lipid oxidation products, which may deteriorate the sensory quality of the produced oil. The aim of the study was to determine the main volatile compounds which differentiate examined oils and could be used as the markers of lipid oxidation in various oils. In the experiment, cold-pressed oils—brown flaxseed, golden flaxseed, hempseed, milk thistle, black cumin, pumpkin, white poppy seed, blue poppy seed, white sesame, black sesame and argan oils from raw and roasted kernels—were analyzed. To induce oxidative changes, an accelerate storage test was performed, and oils were kept at 60 °C for 0, 2, 4, 7 and 10 days. Volatile compound profiling was performed using SPME-GC-HRToFMS. Additionally, basic measurements such as fatty acid composition, peroxide value, scavenging activity and phenolic compound contents were carried out. Multivariate statistical analyses with volatile compound profiling allow us to differentiate oils in terms of plant variety, oxidation level and seed treatment before pressing. Comparing black cumin cold-pressed oil with other oils, significant differences in volatile compound profiles and scavenging activity were observed. Compounds that may serve as indicators of undergoing oxidation processes in flaxseed, poppy seed, milk thistle and hemp oils were determined.

## 1. Introduction

Cold-pressed oils are rich in polyunsaturated fatty acids (PUFA), phenolic compounds, sterols and carotenoids, which are desirable in human nutrition. However, the most remarkable differences between cold-pressed and refined oils are connected with their aroma, taste and color [1,2,3]. While in the course of refining the oil loses the latter attributes, in cold-pressed oils, organoleptic properties are related to the high quality of the product [4]. On the other hand, PUFAs are prone to oxidation, which may cause formation of lipid oxidation products, mainly volatile compounds responsible for deterioration of oil sensory quality manifested as rancid off-flavors.

Volatile compounds are responsible for aroma formation, but not every volatile compound has a characteristic odor. The complexity of flavor is based on the composition of several or more volatiles which have not only various odor thresholds (the lowest concentration, where one can detect the odor of the compound), but they also differ depending on the matrix. Thus, a food product may contain a high concentration of volatiles which have no influence on the aroma quality, and at the same time a very low concentration of the compound which significantly lowers the quality of the oil. Oil aroma may be affected by volatile compounds that have the origin in the plant material or are the effects of processing or storage, such as short chained fatty acids, heterocyclic compounds, ketones, alcohols, esters and aldehydes [5]. Most chemical changes in cold-pressed oils take place during storage, and they are related to the oxidation of unsaturated fatty acids [6]. Therefore, cold-pressed oils should be consumed within 6–12 months after production. The main group of volatiles which have a crucial impact on the deterioration of oil quality comprises lipid oxidation products, e.g., aldehydes, ketones, esters and furan derivatives [7,8]. Several compounds, such as hexanal or nonanal, are used as markers of the lipid oxidation process [9,10].

Volatile compound analysis in food is still a challenge because of the low stability of some analytes and their low concentration in the product. Recently, many isolation techniques have been developed, including solventless techniques, along with conventional extraction or distillation, such as simultaneous distillation extraction (SDE), solvent assisted flavor evaporation (SAFE), static headspace (SHS), headspace sorptive extraction (HSSE) and direct thermal desorption (DTD) [11,12,13]. Those techniques, coupled with gas chromatography and mass spectrometry, facilitate monitoring of volatile compounds in the product. To isolate volatiles, it is recommended to use techniques, which do not require the use of a solvent and high temperature, which may cause the formation of artifacts or lead to degradation of some volatile compounds. The most commonly used isolation technique is solid phase extraction (SPME). SPME is widely applied in routine volatile compound analysis thanks to its simplicity, high sensitivity, linearity and repeatability. SPME, in comparison to other techniques involving solvents, allows us to identify and quantify low molecular volatile compounds, which coelute with the solvent peak during chromatographic analysis [14,15].

Modern food analytics navigates towards the “-omic” approach (foodomics, metabolomics, volatilomics, flavoromics and sensomics). It includes a comprehensive analysis of low molecular compounds, originally found in the product, and those which are secondary products or compounds formed during the technological processes or product storage [16]. Flavoromics is focused on compounds responsible for flavor found in a broad spectrum of analyzed metabolites [17], while sensomics takes into account odor or taste active molecules analyzed with tools involving human senses, as gas chromatography—olfactometry (GC-O) [18]. Such a wide range of analyses requires appropriate tools, which can isolate most analytes and facilitates further identification and quantification. Volatile compound profiling with the use of solid phase microextraction with comprehensive two-dimensional gas chromatography and time of flight mass spectrometry (SPME-GC×GC-ToFMS) provides a detailed untargeted analysis, also focused on the differentiation of samples in terms of their technological processes, variety, origin, etc. [19,20,21]. Compared to GC×GC-ToFMS, GCHRT-MS is characterized by high resolution of mass spectrometry, which is highly recommended in analyses of complex matrices such as food because of selectivity provided by the accurate mass measurement. Additionally, very high sensitivity allows us to identify and quantify compounds which are present in the sample even in trace amounts. Coupled with SPME, it can be used to monitor changes even at the beginning of the quality alteration in the product. Volatile compound profiling provides a large data matrix, which requires multivariate statistical analyses (MVA). The most commonly used MVA in food analytics are principal component analysis (PCA) and partial least squares regression (PLS), which are focused on the determination of volatile compounds responsible for the differentiation between samples. Coupled with SPME-GCHRT-MS, it gives a powerful tool to discriminate products in authenticity testing as well as identifying possible markers of processes and changes undergoing in foods, e.g., lipid oxidation products in oils.

The aim of the study was to apply SPME-GCHRT-MS to differentiate cold-pressed oils based on their volatile compound profiles and to determine the main volatile markers of lipid oxidation process in these oils, which were characterized also by their peroxide value (PV), phenolic compounds and radical scavenging activity (DPPH). The following oils were investigated: golden flaxseed (GF), brown flaxseed (BF), blue poppy seed (BP), white sesame (WS), white poppy seed (WP), argan (RA), roasted argan (ROA), black cumin (BC), pumpkin (RP), hemp (HE), black sesame (BS) and milk thistle (MT).

## 2. Results

### 2.1. Peroxide Value

Peroxide value is an indicator of the oxidation process. In our experiment, peroxide value was measured on day 0, 2, 4, 7 and 10 of the experiment. It was observed (Table 1) that the highest PV on day 0 was recorded in white poppy seed oil (WP) (111.08 meq O_2_/kg), while the lowest PV was found in white sesame oil (WS) (0.43 meq O_2_/kg). In most samples, the highest value of PV was measured at the end of the experiment. The highest PV value reaching 261.94 meq O_2_/kg was detected in WP, while the lowest—in the cold-pressed oil obtained from roasted argan kernels, ROA, reaching 7.07 meq O_2_/kg. In black cumin oil (BC) and WP, the PV value reached the highest values on the 4th (253.12, 357.59 meq O_2_/kg, respectively), while in ROA on the 7th (7.59 meq O_2_/kg) day of storage. Depending on the variety, cold-pressed oil obtained from the same plant differed in PV during the experiment. That phenomenon was observed in black poppy seed oil (BP) and WP, brown flaxseed (BF) and golden flaxseed (GF), as well as WS and BS. Furthermore, thermal processing influenced PV values (RA vs. ROA). Detailed information on PV changes in particular days are provided in Table S1.

### 2.2. Determination of Radical Scavenging Activity

Radical scavenging activity was measured on day 0 and 10 of the experiment (Table 1). The highest value was observed in black cumin (BC) reaching 95.63% AA on day 0 and 95.20% AA on day 10, while the lowest DPPH was determined in blue poppy seed (BP) reaching 18.68 and 7.46% AA, respectively. While DPPH in BC remained constant during the experiment, the average decrease in radical scavenging activity was 50%, where in RP DPPH decreased to 11% and in HE to 75%. No such significant differences between cold-pressed oil obtained from the same plants but different varieties were noticed in DPPH measurement.

### 2.3. Phenolic Compounds

The total content of phenolics was determined at the beginning and at the end of the experiment (Table 1). Additionally, in this measurement, BC obtained the highest values reaching 388.18 and 274.22 mg/kg (0 and 10th day, respectively). RA had the lowest content of phenolic compounds, 23.91 and 11.71 mg/kg. The greatest differences between the samples obtained from the same plant were found in the case of cold-pressed oil produced from sesame; on day 0, WS had a 33% higher content of phenolics than BS (104.06 vs. 69.65 mg/kg, respectively). The highest degradation of the phenolic compounds was observed in RA (51%), while in the ROA sample, no change was observed in the phenolic compound concentrations.

### 2.4. Fatty Acid Composition

The fatty acid composition was analyzed on day 0 and on the last day of the experiment. The measurement was performed with the use of GC-FID. To determine the concentration and the percentage of each fatty acid, the internal standard C19:0 was used. The results are shown in Table S2 in Supplementary Files. The main fatty acids in analyzed cold-pressed oils were oleic acid (C18:1), linoleic acid (C18:2) and linolenic acid (C18:3). Those oils were represented in the highest percentage in ROA (48% of C18:1), WP (73% of C18:2) and GF (61% of C18:3). Flaxseed oils were the samples with the highest abundance of linolenic acid (C18:3). Comparing oils obtained from the same plant, it was observed that BF and GF varied in terms of the percentage of oleic acid (22 and 15%, respectively) and linolenic acid (52 and 61%, respectively). Furthermore, in poppy seed oils, the percentage of linoleic acid was different reaching 14% in WP and 18% in BP. The highest amount of fatty acids in analyzed samples was as follows: oleic acid (C18:1) in argan oils (267 and 298 mg/kg), linoleic acid (C18:2) in poppy seed oils (406 and 390 mg/kg) and linolenic acid (C18:3) in flax seed oils (332 and 359 mg/kg). In most samples, the concentration of the above-mentioned acids decreased during the accelerated storage test. In RA and BP, the amount of oleic, linoleic and linolenic acids was the same at the beginning and the end of the experiment. The average decrease in C18:1 was about 14%, in GF reaching 19%. Oleic acid concentration in WS decreased by 18%, while in the other samples, the value was about 11%. The content of C18:3n3, present in high concentrations in cold-pressed oils obtained from flaxseeds, decreased by about 20% in BF and 10% in G, respectively (considering their mg/mL contents).

### 2.5. Volatile Compound Profiling in Cold-Pressed Oils

**Figure 1:**
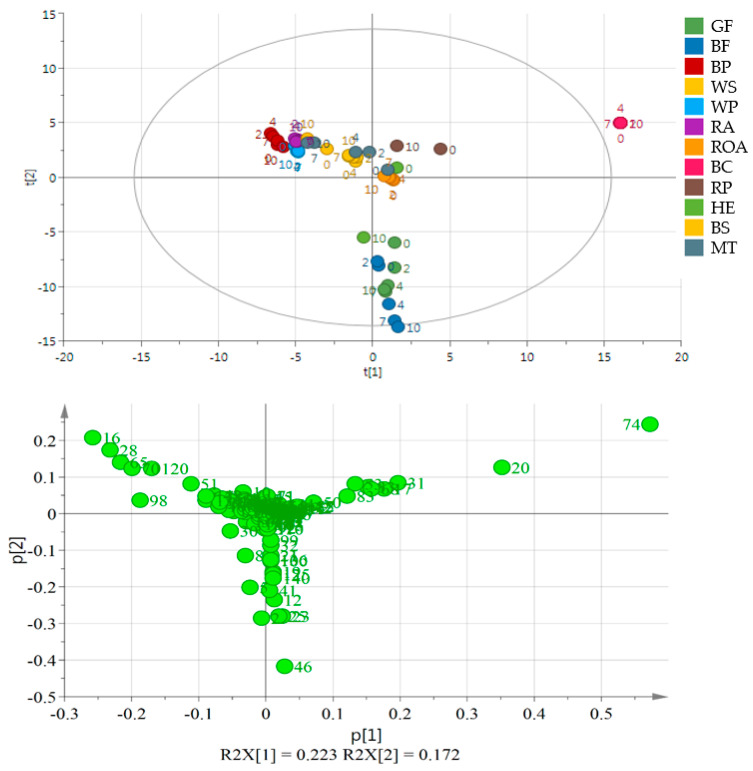
Principal component analysis (PCA) and the loading plot of all analyzed oils stored at 60 °C for 0, 2, 4, 7 and 10 days. On the loading plot compounds responsible for differentiation are placed far from the plot center. **16**—pentanal, **28**—hexanal, **120**—acetic acid, **20**—3-thujene, **46**—1-penten-3-ol, **51**—2-heptanone, **65**—2-pentyl furan, **70**—1-pentanol, **74**—*trans*-β-ocimene, **98**—1-hexanol. Sample coding as in Table S3.

Volatile compound analysis was performed on day 0, 2, 4, 7 and 10 of the experiment using the GC-HRToFMS apparatus. Several oils—RP, HE and BS—were analyzed only on day 0 and the last day of the experiment. The number of identified volatiles selected for Principal Component Analysis (PCA) in samples was 189. The Supplementary Material (Table S3) provides a list of volatile compounds identified. The table shows the most characteristic compounds for analyzed oils, where most of the analytes were lipid oxidation products such as aldehydes, ketones and alcohols (Table S4). To present the differences between the samples, the multivariate statistical analyses were performed using the SIMCA software (ULMETICS, Umeå, Sweden). On the PCA plot (Figure 1), 3 separate clusters are present grouping oils of similar character regarding their profile of volatile compounds—namely, black cumin oil, flaxseed oils and the remaining ones as a third cluster.

**Figure 2:**
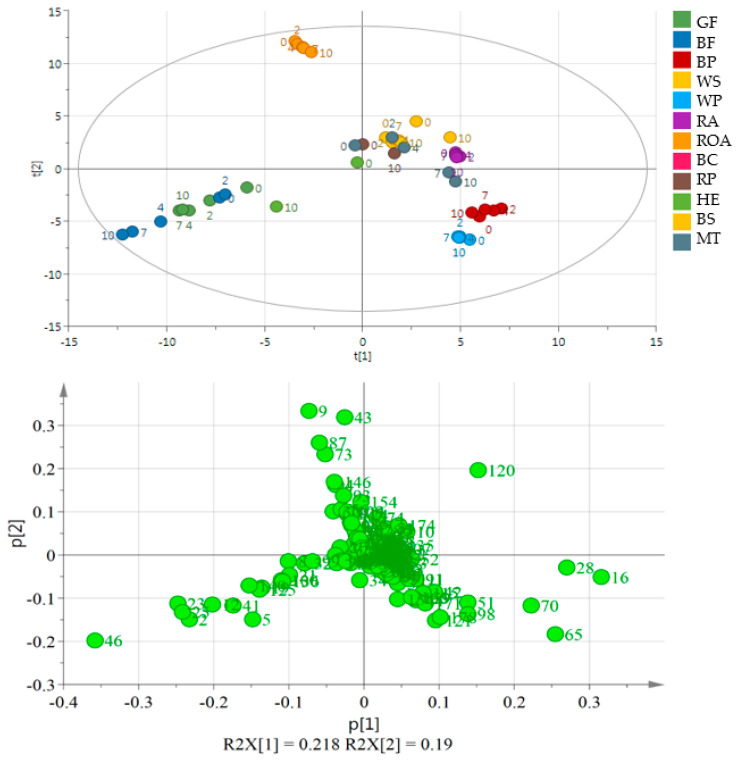
PCA and the loading plot of analyzed oils (except for BC) stored at 60 °C for 0, 2, 4, 7 and 10 days. On the loading plot compounds responsible for differentiation are placed far from the plot center. **2**—propanal, **9**—2-methyl butanal, **16**—pentanal, **20**—3-thujene, **23**—1-propanol, **28**—hexanal, **120**—acetic acid, **43**—1-methyl-1H-pyrrole, **46**—1-penten-3-ol, **51**—2-heptanone, **65**—2-pentyl furan, **70**—1-pentanol, **73**—methyl pyrazine, **74**—*trans*-β-ocimene, **87**—2,3-dimethyl pyrazine. Sample coding as in Table S3.

Oil obtained from black cumin was the most different from the others. It is placed out of the range of the PCA plot making the other oils moderately resolved (Figure 1). Oils in the next well-separated group originated from flaxseeds of two varieties. Other samples were placed near the center of the plot or in its 4th quarter (WS and BP, RA, HE). Although black cumin and flaxseed oils were well separated from the other oils, no separation may be indicated on the basis of the day of the experiment or the variety of the used seed (GF and BF). The second PCA plot (Figure 2) shows a very good separation of flaxseed and poppy seed oils from the other samples as they form a distant cluster. Moreover, BP and WP are separated from each other, while GF and BF are mixed. Additionally, ROA as the oil obtained from roasted kernels is well separated, mainly because of the presence of 1-methyl-1H-pyrrole, 2,5-dimethyl- and methyl pyrazine and 2-methyl butanal. During storage, they behaved similarly resulting in a significant increase in the propanal peak area. In ROA also pentanal, hexanal, heptanal and hexanal increased their peak areas almost 2-fold, whereas in RA, the levels of the latter compounds remained constant or increased very slightly. A comparison between RA and ROA was published in an earlier work [22]. All compounds projected on loadings graphs with their numbers are listed in Table S3 in Supplementary Files and their changes in time in Table S4.

In the 1st quarter, sesame oils were placed, but WS was within a very small distance from RP and MT samples. The elimination of BC samples resulted in an improved separation within the obtained data matrix. The first PCA quarter contained WS, RP, RA and BS samples from the whole experiment. MT samples taken from the first days of the storage were placed in the first and the fourth PCA quarters. Those samples, which were placed far from the center, had higher peak areas of acetic acid. WP and BP had a good separation because of the presence of compounds such as pentanal, 2-pentyl furan, hexanal and 1-pentanol. In both oils obtained from poppy seed pentanal, 2-pentyl furan, hexanal, 1-hexanol and 1-octen-3-ol were abundant. Moreover, WP contained significantly more volatile compounds than BP, with 2-pentanone, 2-heptanone, 1-pentanol and hexanoic acid as the most abundant.

**Figure 3:**
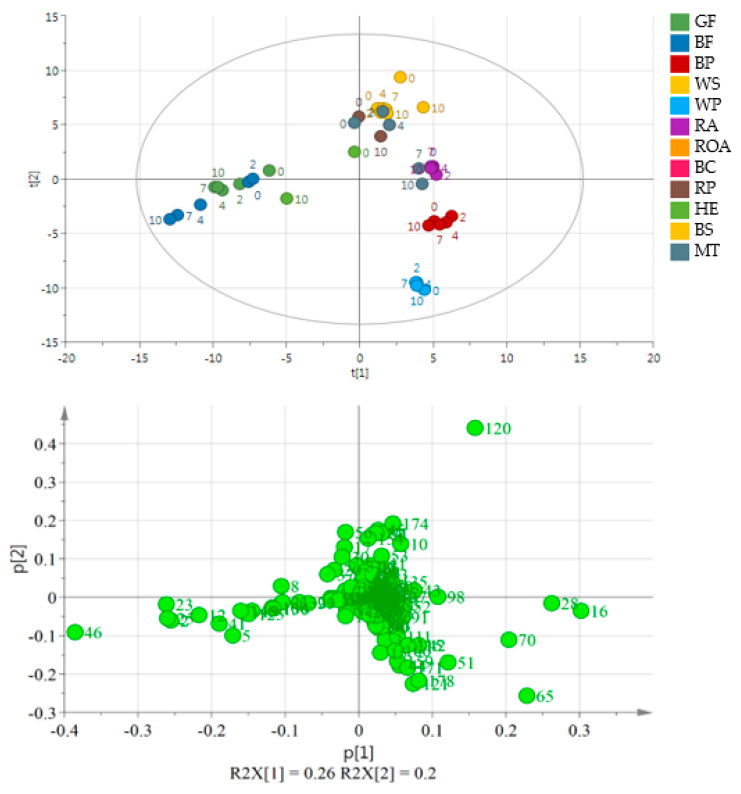
PCA and the loading plot of analyzed oils (except for BC and ROA) stored at 60 °C for 0, 2, 4, 7 and 10 days. On the loading plot compounds responsible for differentiation are placed far from the plot center. **2**—propanal, **12**—2-ethyl furan, **16**—pentanal, **23**—1-propanol, **25**—2-butenal, **28**—hexanal, **46**—1-penten-3-ol, **65**—2-pentyl furan, **70**—1-pentanol, **120**—acetic acid. Sample coding as in Table S3.

Figure 3 shows the results without BC and ROA samples. A significant separation between the two varieties of flaxseed and poppy seed oils may be observed. During storage, the concentrations of the latter oil compounds, except for hexanal and pentanal, increased. Hexanal reached the maximum area peak on the 7th day in BP and the 2nd day in WP. Pentanal also showed the maximum peak on the 2nd day of storage. On the last day of the experiment, an almost 10-fold increase in the hexanal peak area was observed in MT. Additionally, 1-octen-3-ol and 2-heptenal peak areas significantly increased (2- and 3-fold, respectively). In cold-pressed milk thistle oil, the main group of volatile compounds comprised products of lipid oxidation, in particular aldehydes such as butanal, propanal and 2-methyl butanal. The rate of the increase varied for each compound. While propanal and pentanal had the highest peak area on the 7th day of the experiment, 2-methyl butanal was the most abundant on the 4th day. The oils obtained from raw pumpkin and hemp were placed in the center of the PCA plot, mainly because they were analyzed only as fresh and oxidized samples.

**Figure 4:**
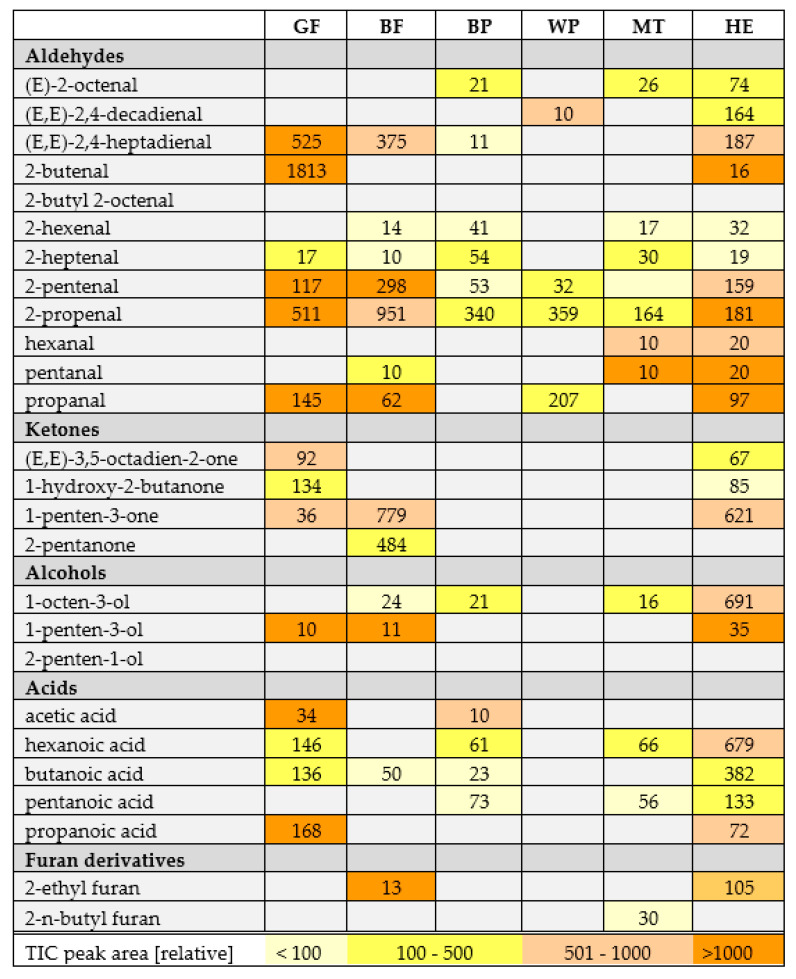
Tabular presentation of most important lipid oxidation products in examined oils. Numbers in cells for particular compounds indicate how many times their concentration (expressed as TIC peak area) increased between day 0 and day 10 of accelerated storage test. To illustrate peak abundances, cells were color-coded (the legend in a last row) to inform what was the area of peak detected on day 10. Sample coding as in Table S3.

Oxidative changes during storage can be observed on Figure 2 and Figure 3 in the case of flaxseed oil of both varieties, but also for hemp oil (all these oils did not form a uniform cluster, but the distances between particular storage days were distinguishable). Figure 4 helps to visualize the changes in volatile compounds for those oils in which changes were most significant. Compounds were classified into main groups of volatile lipid oxidation products. It summarizes results for 6 oils in which the changes were most significant. For the remaining oils, no tenfold or more increase in the peak areas of particular compounds was noted, which was a criterion for selecting autooxidation volatile markers. Figure 4 shows numbers in particular cells indicating how many times the peak area for a given compound increased during accelerated storage (Schaal Oven Test) experiment. The cells are also color-coded indicating the size of the peaks of particular compounds. As a non-targeted approach was used for the analysis of volatile compounds, TIC peak areas were compared. From Figure 4, compound characteristics for particular oil can be selected, which were represented by peaks of the biggest area, as well as the most pronounced increase during accelerated storage test. The most radical increase in lipid oxidation products was noted for flaxseed oil (GF and BF) and also for Hemp oil (HE). To lesser extend the increase in volatile compounds was noted for poppy seed oils (BP, WP) and milk thistle (MT). Interestingly, hexanal was not the best marker of oxidation for oils shown in Figure 4. The highest amount of C18:3 in flaxseed oils and in hemp oil resulted in their rapid oxidation in the test, with a dominating unsaturated aldehydes (2-alkenals and 2,4-alkadienals) share in volatile compounds.

## 3. Discussion

The oxidation process is among the most significant mechanisms influencing the sensory properties of food. In food, the ongoing oxidation process may be observed, e.g., based on the increasing peroxide value or changes in the fatty acid composition. In this study, in all analyzed samples, PV was higher at the end of the experiments compared to the beginning. In the case of cold-pressed oils obtained from hemp and black cumin, the peroxide value was higher even before the storage experiment than the limit specified in Codex Alimentarius [23]. Moreover, in comparison to other studies [24,25] PV in HE was higher, reaching 14.47 meq O_2_/kg (compared to 4.10 and 6.42 meq O_2_/kg). Depending on the variety of the plant seed, results during the accelerated storage varied, as observed to in the case of BP and WP, GF and BF and WS and BS. Furthermore, a comparison of RA with ROA indicated the influence of roasting on the stability of the oil. PV results from the other oils, including MT, in most cases, were similar to those reported in other studies [5,26,27,28].

Cold-pressed oils are valuable sources of unsaturated fatty acids. On the other hand, PUFAs take part in the oxidation process as substrates, resulting, e.g., in the formation of volatile compounds. Additionally, the high concentration of α-linolenic acid (C18:3n-3) is highly desirable because of the potential conversion to the longer-chain n-3 PUFA as EPA and DHA [29]. HE oil is known to have a very good 3:1 ratio of omega-6 to omega-3 fatty acids, which is preferable in human nutrition; however, its high PUFA concentration is related to an advanced oxidation process [30]. The content of α-linolenic acid due to its high susceptibility to autooxidation is a source of a high number and considerable amounts of volatile compounds, especially unsaturated aldehydes.

Volatile compounds are the main group of lipid oxidation products. Some of them are responsible for the deterioration of the product, in particular by changing food aroma. The aim of this study was to determine volatile compounds which may be used as markers for lipid oxidation in selected cold-pressed oils obtained from various plant seeds, as well as show differentiation between particular oils. The results of multivariate statistical analyses showed that BC is the most remarkable oil among the analyzed samples, regardless of the oxidation process. The main compounds responsible for the differences between the BC and the other oils were terpenes such as 3-thujene, trans-β-ocimene, β-pinene, α-pinene, γ-terpinene, sabinene, d-limonene and cis-4-methoxy thujane. Elimination of BC from the data set resulted in better separation (larger distances between clusters). To compare all the oils without any treatment other than the oil pressing, ROA was also removed. The third PCA plot (Figure 3) shows a separation between varieties of flaxseed oils and also poppy seed oils. The main volatile products responsible for separation in those samples are the lipid oxidation products. Compared to BP, WP contains a higher number of volatiles, in particular several furan derivatives which are not present in BP and are known for their high influence on sensory properties of food. Those compounds are the main products of the unsaturated fatty acid autooxidation process. The results are very similar to those reported in other articles [31,32,33,34,35]. Furthermore, Kopuncová et al. [36] identified pentanal, hexanal, 1-octen-3-ol, hexanoic acid and 2-pentyl furan as some of the key odorants in poppy seed. GF and BF oils were placed in the third quarter of the plot with 1-penten-3-ol, 1-propanol, 2-butenal, propanal and 2-ethyl furan as the possible markers of storage. To date, no research on volatiles in milk thistle cold-pressed oil has been published. WS contains a higher number of volatile compounds than BS, probably because of the more advanced oxidation process. Moreover, this black sesame variety contains more terpenes, which are known as antioxidants. The main compounds were lipid oxidation products, but a rapid increase in contents of some compounds was also observed in HE, in particular 2-methyl propanal and (*E*,*E*)-2,4-heptadienal, whose peak areas increased 187-fold.

Untargeted profiling of volatile compounds in cold-pressed oils by SPME-GC-HRToFMS allows the differentiation of some of the oils based on their volatile compound profiles after PCA analysis. Oils with unique flavor significantly influenced by terpenes (as in black cumin) and produced form roasted seeds (ROA—roasted argan) have significantly different profile of volatiles, which makes them distant to the remaining oils analyzed. Profiling volatiles can also visualize changes related to oxidation processes, especially in oils rich in polyunsaturated fatty acids (especially C18:3), such as flaxseed and hemp. However, profiles of the remaining oils—sesame, pumpkin, milk thistle and argan (unroasted)—were highly similar, which resulted in their indistinguishable location in one PCA quarter. Based on the most intense increase in hydroperoxide decomposition products during an accelerated storage test, such compounds as (*E*,*E*)-2,4-heptadienal, 2-pentenal, 2-propenal, propanal 1-penten-3-one, as well as 1-penten-3-ol can be used as indicators of oxidative changes in C18:3 rich oils.

## 4. Materials and Methods

### 4.1. Oils and Accelerated Storage (Schaal Oven Test) Conditions

Cold-pressed oils, brown flaxseed, golden flaxseed, hempseed, milk thistle, black cumin, pumpkin, white and blue poppy seed, as well as white and black sesame, were cold-pressed using a Farmet Uno cold-pressing machine (Farmet, Česká Skalice, Czech Republic) from seeds bought from a local seller. Argan oils, raw and roasted, were obtained from Marokosklep, Poland (USDA-NOP certified organic by ECOCERT S.A., Toulouse, France). The 4.40 g samples of analyzed oils were placed in a closed 44 mL glass vial and kept in an oven at 60 °C for 0, 2, 4, 7 and 10 days. All the samples were stored in a freezer at −70 °C until analyses.

### 4.2. Peroxide Value

Peroxide value analysis was performed in accordance with PN-EN ISO 3960:2012 based on iodometric (visual) determination of the end point of titration.

### 4.3. Determination of Radical Scavenging Activity

Radical scavenging activity was measured using an Epoch Microplate Spectrophotometer (BioTek, Winooski, VT, USA). For this purpose, DPPH solution was prepared by diluting 52 mg of DPPH in 100 mL of pure methanol. The solution was diluted ten times. For the calibration curve, 52 mg of ascorbic acid was dissolved in 50 mL of water and diluted to obtain various concentration levels. The samples were prepared by diluting 1 g of oil in 3 mL of a methanol and water (80:20 *v*/*v*) mixture and shaking for 3 min. The 100 µL samples were placed on the reader plate; then, each was supplemented with 100 µL diluted DPPH solution. The reader plate was slightly shaken and placed in a dark place for 15 min. After this time, the absorbance was read at 517 nm. The calibration curve was prepared in the same way using an ascorbic acid solution in various concentrations instead of samples.

### 4.4. Determination of PHENOLIC Compounds

Concentrations of phenolic compounds were measured with the use of a 96 microplate reader (Epoch Microplate Spectrophotometer). The calibration curve was prepared using the Folin-Ciocalteu reagent (FCS) diluted in water at various concentrations. An amount of 1 mg of phloroglucinol solution was diluted in 1 mL of 80% methanol. Samples were prepared in the same way as in the radical scavenging activity measurement. An amount of 1 g of oil was diluted in 3 mL of methanol and water (80:20 *v*/*v*) mixture and shaken. An amount of 100 µL of sample was placed on a microplate, then 165 µL of FCS was added and left for 3 min. The next step was to add 60 µL 2 M sodium carbonate and 80 µL water. The microplate was slightly shaken and placed in a dark place for 60 min. After incubation, the absorbance of the samples was measured at 725 nm. The concentration of phenolic compounds was calculated based on the phloroglucinol absorbance value.

### 4.5. GC-FID Analysis of Fatty Methyl Esters

Fatty acid composition was determined using GC-FID (Agilent Technologies 6890N GC System, Willmington, DE, USA). For this purpose, to one weighed drop of the analyzed oil, 50 µL of the internal standard C19:0 was added. After 10 min, 0.5 mL of NaOH (in methanol) was added. The mixture was placed in a heater at 80 °C for 30 min. After cooling, 200 µL of BF3 and 1 mL of saturated NaCl were added. The mixture was shaken, and the heptane layer was transferred to a 1.5mL glass vial. Fatty acid composition was determined using GC-FID with the column SP2560 (95 m × 0.25 mm × 0.2 µm) and split 1:100. The oven program was as follows: the initial temperature was 140 °C for 5 min, then 4 °C/min to 240 °C. The identification of fatty acids was performed on the basis of retention times of fatty acid standards. The concentration and the percentage of analytes was calculated using the C19:0 standard (Sigma Aldrich, Prague, Czech Republic).

### 4.6. SPME-GC-HRT Volatile Compound Profiling in Cold-Pressed Oils

Carboxene/divinylbenzene/polydimethylsiloxane (CAR/DVB/PDMS) 1 cm fiber was used (Supelco, Bellefonte, PA, USA). An amount of 250 mg of oil was placed in a 10 mL glass vial and closed with a silicone seal. The sample was incubated at 40 °C for 5 min. The extraction of the sample lasted 10 min. The analysis of volatile compounds was performed with the use of an Agilent 7890A gas chromatograph (Agilent Technologies, Wilmington, DE, USA), equipped with a multifunctional MPS 2 autosampler (Gerstel, Switzerland) and coupled with a Pegasus HRT (LECO, St. Joseph, MI, USA) ultrahigh resolution time of flight mass spectrometer (resolving power 25 000 FWHM). The gas chromatograph was equipped with the HP-Innowax column (30 m × 0.25 mm × 0.25 µm). Analyses were performed in the splitless mode. The injection port temperature was 270 °C and constant flow 1 mL/min. Purge time was 120 s. The oven program was the following: the initial temperature 40 °C for 2 min, then 6 °C/min to 130 °C, followed by 30 °C/min to 270 °C and held for 2 min. The total GC run time was 22.5 min. Data processing such as automatic peak find, spectral deconvolution and compound identification was performed using ChromaTOF (LECO, St. Joseph, MI, USA). Data were collected in the mass range of 35–510 Da. Full spectra information was collected at a speed of 5 Hz.

### 4.7. Statistical Analysis

The collected data were deconvoluted and identified by comparing the obtained spectra with the NIST spectral library using the ChromaToF software. For the purpose of statistical analyses, peaks with a signal to noise ratio higher than 100 were manually selected for each sample. All areas of the selected peaks were normalized and analyzed using the SIMCA 14.0 software (Umetrics, Umea, Sweden). To visualize differences between the oils, the principal component analysis was performed. This statistical method allowed us to select the most important markers from volatile fingerprints for each oil.

## 5. Conclusions

Multivariate statistical analyses with volatile compound profiling indicates differences between cold-pressed oils depending on plant variety, oxidation level and seed treatment before pressing. When comparing BC with other oils, significant differences in volatile compound profiles and scavenging activity were observed. This was probably caused by the presence of the high number of terpenes in BC oil. Cold-pressed oil obtained from roasted argan kernels was characterized by the presence of pyrazines and pyrroles, which are mainly related to the Maillard reaction. The analytical tools used may be successfully applied to monitor the quality of cold-pressed oils focusing on the presence of lipid oxidation products and to differentiate them based on the plant origin.

## Figures and Tables

**Table 1:** Peroxide value (PV) [meq O_2_/kg], phenolic compound contents [mg/kg] and radical scavenging activity (DPPH) [AA%] in analyzed oils before (F) and after storage at 60 °C for 10 days (O). GF—golden flaxseed, BF—brown flaxseed, BP—blue poppy seed, WS—white sesame, WP—white poppy seed, RA—argan, ROA—roasted argan, BC—black cumin, RP—pumpkin, HE—hemp, BS—black sesame, MT—milk thistle.

Sample		PV [meq O_2_/kg]	Phenolic Compounds [mg/kg]	DPPH [AA%]
BF	F	0.56 ± 0.02	30.82 ± 0.83	21.41 ± 0.51
	O	103.57 ± 0.97	22.59 ± 0.00	10.75 ± 0.18
GF	F	0.67 ± 0.02	28.59 ± 0.88	26.64 ± 0.20
	O	84.30 ± 0.39	22.59 ± 0.83	11.31 ± 1.79
RA	F	6.12 ± 0.08	23.91 ± 0.44	21.41 ± 0.41
	O	43.66 ± 0.45	11.71 ± 2.91	9.04 ± 2.23
ROA	F	1.92 ± 0.06	23.91 ± 0.22	22.70 ± 2.84
	O	7.07 ± 0.11	23.47 ± 2.91	11.88 ± 1.34
WP	F	111.08 ± 0.79	34.84 ± 1.25	22.77 ± 0.10
	O	261.94 ± 0.74	35.82 ± 2.91	10.24 ± 0.80
BP	F	0.68 ± 0.06	31.41 ± 0.44	18.68 ± 0.10
	O	100.14 ± 0.81	24.94 ± 3.33	7.46 ± 1.43
WS	F	0.43 ± 0.09	104.06 ± 1.25	27.36 ± 0.20
	O	11.26 ± 0.08	85.00 ± 5.52	13.40 ± 1.79
BS	F	0.75 ± 0.04	69.65 ± 0.83	31.45 ± 0.91
	O	18.14 ± 0.22	61.25 ± 1.99	18.46 ± 0.36
MT	F	4.00 ± 0.02	59.22 ± 3.98	34.68 ± 1.22
	O	135.49 ± 1.40	57.00 ± 1.25	18.52 ± 1.25
BC	F	68.83 ± 0.09	388.18 ± 12.89	95.63 ± 0.10
	O	206.69 ± 0.54	274.22 ± 7.07	95.20 ± 0.18
HE	F	14.47 ± 0.43	51.41 ± 7.07	33.67 ± 2.23
	O	153.98 ± 0.98	49.06 ± 4.16	8.22 ± 0.72
RP	F	18.76 ± 0.46	101.88 ± 1.10	24.13 ± 0.41
	O	92.52 ± 1.66	99.94 ± 1.62	21.49 ± 2.23

## Data Availability

The data presented in this study is available in supplementary material.

## Supplementary Materials

The following are available online: Tables S1–S4.

## Sample Availability

Samples are not available from the authors.
