# Supplementary material for: Changes in Volatile Compound Profiles in Cold-Pressed Oils Obtained from Various Seeds during Accelerated Storage
Source: Molecules. 2021 Jan 8;26(2):285. doi: 10.3390/molecules26020285 (PMC7827483; doi:10.3390/molecules26020285)
Supplement: Supplementary file 1 [file molecules-26-00285-s001.pdf]

Table 1S. Fatty acid composition [%, w/w] and their contents [mg/ml] in analyzed oils before (**F**) and after (**O**) accelerated storage test performed at 60 °C for 10 days. Sample coding as in Table 1.

												%											
		C8:0	C12:0	C14:0	C15:0	C16:0	C16:1	C17:0	C18:0	C18:1	C18:2	C18:3	C20:0	C20:1	C20:2	C20:3	C20:5	C22:0	C22:1	C22:2	C22:6	C24:0	C24:1
BF	F			0,03	0,02	4,80	0,06	0,06	4,43	22,22	15,31	52,32	0,21	0,16	0,02	0,03		0,16	0,04			0,12	0,01
	O			0,03	0,02	4,71	0,06	0,06	4,51	22,54	15,34	51,92	0,21	0,18	0,03	0,03		0,17	0,05			0,13	0,02
GF	F			0,02	0,01	4,05	0,05	0,06	3,71	14,71	15,66	60,75	0,18	0,14	0,04	0,23		0,18	0,09			0,10	0,01
	O			0,03	0,01	4,04	0,05	0,06	3,70	14,75	15,68	60,70	0,18	0,14	0,02	0,24		0,18	0,10			0,10	0,02
RA	F			0,12	0,04	11,91	0,09	0,08	6,13	45,83	34,14	0,19	0,54	0,42	0,03	0,20	0,01	0,15	0,02			0,08	0,02
	O			0,11	0,04	11,27	0,08	0,08	6,28	46,39	33,99	0,16	0,57	0,43	0,04	0,22	0,02	0,16	0,03			0,08	0,04
ROA	F			0,11	0,04	11,56	0,10	0,08	5,70	47,69	33,16	0,15	0,53	0,45	0,04	0,06		0,17	0,02			0,09	0,03
	O			0,11	0,04	11,10	0,10	0,08	5,76	47,58	33,48	0,15	0,53	0,45	0,05	0,26		0,17	0,03			0,08	0,03
WP	F	0,01		0,05	0,01	8,31	0,13	0,06	2,78	13,58	73,46	0,55	0,42	0,08	0,08		0,22	0,03	0,03	0,02	0,03	0,03	0,14
	O	0,02		0,04	0,02	8,41	0,13	0,06	2,93	13,98	72,63	0,53	0,42	0,09	0,09		0,31	0,03	0,04	0,00	0,02	0,03	0,22
BP	F			0,04	0,02	7,84	0,12	0,06	2,08	17,68	70,81	0,71	0,36	0,10	0,05		0,05	0,03				0,03	0,04
	O			0,03	0,01	7,77	0,11	0,05	2,05	17,50	71,28	0,72	0,27	0,10	0,04			0,02				0,03	0,01
WS	F			0,01		9,23	0,13	0,06	6,13	42,50	40,20	0,31	0,90	0,19	0,02		0,01	0,17				0,12	
	O			0,02		8,84	0,13	0,06	6,15	42,83	40,18	0,32	0,95	0,20			0,01	0,18				0,13	
BS	F			0,01		9,13	0,14	0,05	5,51	40,16	43,35	0,26	0,84	0,20	0,05			0,17				0,12	
	O			0,01		8,51	0,13	0,05	4,75	40,37	44,51	0,28	0,90	0,20	0,01			0,17				0,12	
MT	F			0,08	0,02	7,35	0,06	0,08	5,07	24,13	54,22	0,22	3,67	1,12	0,06		0,02	2,93	0,05			0,88	0,06
	O			0,07	0,02	7,34	0,06	0,08	5,06	24,09	54,10	0,20	3,76	1,12	0,07		0,08	2,95	0,05			0,87	0,07
BC	F			0,16	0,03	10,88	0,19	0,06	3,08	24,11	57,54	0,25	0,30	0,39	2,86		0,02	0,04		0,05		0,03	0,01
	O			0,15	0,03	11,09	0,20	0,06	3,09	24,19	57,35	0,25	0,18	0,40	2,88			0,04		0,05		0,03	0,00
HE	F			0,03	0,01	6,08	0,11	0,06	3,06	14,89	57,53	16,05	0,99	0,36	0,29	0,03	0,02	0,32	0,02			0,15	0,03
	O			0,03	0,01	6,36	0,10	0,06	3,18	15,21	57,30	15,54	1,01	0,37	0,28	0,02	0,02	0,33	0,02			0,15	0,03
RP	F		0,01	0,08	0,01	9,93	0,09	0,09	6,15	32,92	49,00	0,28	0,69	0,15	0,06	0,07	0,03	0,18	0,05			0,11	0,09
	O		0,01	0,08		9,43	0,09	0,09	6,04	32,73	49,56	0,32	0,69	0,15	0,05	0,25	0,09	0,19	0,04			0,10	0,09

mg/ml

		C8:0	C12:0	C14:0	C15:0	C16:0	C16:1	C17:0	C18:0	C18:1	C18:2	C18:3	C20:0	C20:1	C20:2	C20:3	C20:5	C22:0	C22:1	C22:2	C22:6	C24:0	C24:1
BF	F			0,21	0,12	30,53	0,35	0,38	28,13	141,22	97,29	332,46	1,33	1,03	0,15	0,16		1,00	0,27			0,76	0,06
	O			0,16	0,10	23,94	0,29	0,32	22,93	114,64	78,04	264,07	1,09	0,90	0,13	0,13		0,87	0,26			0,68	0,09
GF	F			0,15	0,08	23,95	0,27	0,37	21,93	86,97	92,58	359,08	1,05	0,83	0,22	1,34		1,04	0,55			0,60	0,08
	O			0,15	0,07	21,39	0,26	0,33	19,60	78,22	83,11	321,82	0,95	0,76	0,12	1,26		0,96	0,53			0,54	0,10
RA	F			0,70	0,26	69,43	0,53	0,49	35,73	267,25	199,07	1,09	3,15	2,44	0,19	1,18	0,05	0,88	0,12			0,47	0,12
	O			0,67	0,25	67,48	0,48	0,50	37,59	277,82	203,52	0,94	3,42	2,60	0,24	1,32	0,14	0,97	0,16			0,51	0,24
ROA	F			0,69	0,27	72,28	0,63	0,51	35,61	298,10	207,31	0,95	3,34	2,84	0,24	0,40		1,07	0,15			0,54	0,17
	O			0,55	0,22	57,59	0,53	0,40	29,89	246,80	173,66	0,80	2,72	2,33	0,26	1,33		0,89	0,13			0,43	0,15
WP	F	0,08		0,26	0,08	45,97	0,70	0,32	15,39	75,16	406,56	3,03	2,30	0,43	0,44		1,24	0,15	0,16	0,08	0,15	0,14	0,78
	O	0,11		0,20	0,07	37,92	0,57	0,28	13,21	63,02	327,47	2,37	1,89	0,40	0,39		1,39	0,16	0,19		0,08	0,13	1,01
BP	F			0,18	0,08	42,57	0,62	0,29	11,21	95,78	390,24	3,94	1,50	0,55	0,21			0,12				0,14	0,05
	O			0,21	0,09	43,50	0,65	0,31	11,55	98,09	392,91	3,93	2,02	0,58	0,28		0,27	0,15				0,15	0,20
WS	F			0,08		50,77	0,69	0,34	33,68	233,64	220,99	1,71	4,95	1,07	0,11		0,08	0,96				0,68	
	O			0,07		40,90	0,58	0,28	28,43	198,11	185,85	1,48	4,37	0,92			0,06	0,84				0,60	
BS	F			0,07		50,93	0,77	0,29	30,76	223,99	241,77	1,46	4,68	1,14	0,25			0,93				0,67	
	O			0,06		41,2	0,65	0,23	22,98	195,43	215,49	1,34	4,35	0,95	0,07			0,83				0,57	
MT	F			0,46	0,10	44,28	0,33	0,49	30,54	145,44	326,81	1,31	22,14	6,74	0,35		0,12	17,66	0,29			5,33	0,37
	O			0,38	0,09	39,30	0,32	0,43	27,10	128,93	289,56	1,09	20,13	6,00	0,38		0,42	15,80	0,25			4,65	0,36
BC	F			0,95	0,17	66,11	1,16	0,37	18,75	146,51	349,69	1,53	1,82	2,40	17,36		0,10	0,26		0,31		0,19	0,07
	O			0,79	0,16	59,75	1,08	0,32	16,63	130,40	309,10	1,33	0,97	2,16	15,53		0,02	0,24		0,29		0,17	0,01
HE	F			0,18	0,07	36,77	0,64	0,34	18,48	89,99	347,75	97,03	5,98	2,17	1,75	0,17	0,10	1,93	0,11			0,89	0,13
	O			0,15	0,06	33,35	0,55	0,30	16,71	79,83	300,64	81,54	5,29	1,94	1,47	0,11	0,09	1,74	0,08			0,79	0,05
RP	F		0,06	0,45	0,07	55,56	0,52	0,52	34,46	184,33	274,38	1,59	3,85	0,87	0,33	0,38	0,16	1,03	0,26			0,61	0,48
	O		0,06	0,35		42,26	0,42	0,41	27,08	146,73	222,21	1,41	3,09	0,67	0,24	1,14	0,39	0,87	0,18			0,44	0,42

Table 2S. List of compounds identified in examined oils based on high resolution mass spectrometry.

Compound number	Rt [min]	Compound name	Base Peak Ion exact mass
1	1.44667	Dimethyl sulfide	BPI(62.018371±3ppm)
2	1.57035	Propanal	BPI(58.041310±3ppm)
3	1.59667	Octane	BPI(43.054255±3ppm)
4	1.63333	Propanal, 2-methyl-	BPI(43.054242±3ppm)
5	1.80525	2-Propenal	BPI(56.02573±5ppm)
6	1.93	2-Octene, (Z)-	BPI(55.054257±3ppm)
7	1.95851	Butanal	BPI(44.025618±3ppm)
8	2.13333	2-Butanone	BPI(43.018042±3ppm)
9	2.21333	Butanal, 2-methyl-	BPI(41.038694±3ppm)
10	2.26044	Butanal, 3-methyl-	BPI(44.025804±3ppm)
11	2.45611	Benzene	BPI(78.046500±3ppm)
12	2.58	Furan, 2-ethyl-	BPI(81.033555±3ppm)
13	2.59094	1,3-Octadiene	BPI(54.046471±3ppm)
14	2.83	2,3-Butanedione	BPI(43.017972±3ppm)
15	2.81667	2-Pentanone	BPI(86.07230±3ppm)
16	2.83312	Pentanal	BPI(44.025764±3ppm)
17	3.32667	à-Pinene	BPI(93.069858±3ppm)
18	3.24	Isobutyl acetate	BPI(43.017965±3ppm)
19	3.34	1-Penten-3-one	BPI(55.017879±3ppm)
20	3.39476	3-Thujene	BPI(93.069895±3ppm)
21	3.4	2-Butanol	BPI(43.01797±3ppm)
22	3.47667	Furan, 2-propyl-	BPI(81.033615±3ppm)
23	3.58667	1-Propanol	BPI(31.017930±3ppm)
24	3.6	Oxirane, butyl-	BPI(71.049174±3ppm)
25	3.62	2-Butenal	BPI(41.038735±3ppm)
26	3.89333	Camphene	BPI(67.05425±3ppm)
27	4.03667	Disulfide, dimethyl	BPI(93.990563±3ppm)
28	4.24333	Hexanal	BPI(72.05720±5ppm)
29	4.28667	Butanenitrile, 2-methyl-	BPI(55.041674±3ppm)
30	4.40333	1-Propanol, 2-methyl-	BPI(43.054316±3ppm)
31	4.54333	betapinen	BPI(93.069869±3ppm)
32	4.67698	3-Pentanol	BPI(59.049140±3ppm)
33	4.81333	Sabinene	BPI(93.069898±3ppm)
34	4.90106	2-Pentanol	BPI(45.033635±3ppm)
35	4.93333	1-Butanol, 3-methyl-, acetate	BPI(43.017943±3ppm)
36	4.94	Formic acid, pentyl ester	BPI(55.054281±3ppm)
37	4.93	Ethylbenzene	BPI(91.054237±3ppm)
38	4.96667	Butanenitrile, 3-methyl-	BPI(43.054366±3ppm)
39	4.98	2-Propanol, 1-methoxy-	BPI(45.033579±3ppm)
40	5.02	Bicyclo[3.1.0]hex-2-ene, 4-methylene-1-(1-methylethyl)-	BPI(91.054294±3ppm)
41	5.05333	2-Pentenal	BPI(55.054273±3ppm)
42	5.11788	2-n-Butyl furan	BPI(81.033617±3ppm)

43	5.22667	1H-Pyrrole, 1-methyl-	BPI(81.057335±3ppm)
44	5.30667	Oxirane, pentyl-	BPI(71.049175±3ppm)
45	5.34285	2-carene	BPI(93.069856±3ppm)
46	5.63	1-Penten-3-ol	BPI(57.033501±3ppm)
47	5.64333	3-Carene	BPI(93.069866±3ppm)
48	5.70048	à-Phellandrene	BPI(93.069934±3ppm)
49	5.92	Acetic acid, pentyl ester	BPI(43.017976±3ppm)
50	5.92667	á-Myrcene	BPI(121.101249±3ppm)
51	6.06333	2-Heptanone	BPI(43.018003±3ppm)
52	6.12667	Heptanal	BPI(44.025790±3ppm)
53	6.32031	D-Limonene	BPI(68.062071±3ppm)
54	6.38909	1-Hepten-3-one	BPI(55.017889±3ppm)
55	6.48334	Eucalyptol	BPI(71.04912±5ppm)
56	6.54667	1-Butanol, 3-methyl-	BPI(41.038764±3ppm)
57	6.57564	1,3-Diazine	BPI(80.036963±3ppm)
58	6.6	(R)-(+)-3-Methylcyclopentanone	BPI(69.033584±3ppm)
59	6.70028	3-Hexen-2-one	BPI(83.049235±3ppm)
60	6.75667	2-Hexenal	BPI(83.04913±3ppm)
61	6.78667	Furan, 3-methyl-	BPI(81.033601±3ppm)
62	6.85333	2-Hexanol	BPI(45.033595±3ppm)
63	6.88333	Formic acid, hexyl ester	BPI(56.062090±3ppm)
64	6.93333	1-Methoxy-2-propyl acetate	BPI(43.017974±3ppm)
65	7.05896	Furan, 2-pentyl-	BPI(81.033601±3ppm)
66	7.13	ß-cis-Ocimene	BPI(93.069801±3ppm)
67	7.18	Propanoic acid, 2-oxo-, methyl ester	BPI(43.017970±3ppm)
68	7.26667	ç-Terpinene	BPI(93.069823±3ppm)
69	7.36333	Thiazole	BPI(84.998108±3ppm)
70	7.43333	1-Pentanol	BPI(42.046544±3ppm)
71	7.45667	cis-4-methoxy thujane	BPI(93.069871±3ppm)
72	7.52	3-Octanone	BPI(57.033515±3ppm)
73	7.70667	Pyrazine, methyl-	BPI(94.052467±3ppm)
74	7.80333	trans-ß-Ocimene	BPI(119.085527±3ppm)
75	7.8482	Methallyl cyanide	BPI(41.038712±3ppm)
76	7.905	Furan, 3-pentyl-	BPI(82.041274±3ppm)
77	8.03	Terpinolen	BPI(93.069867±3ppm)
78	8.04667	Thiazole, 2,4-dimethyl-	BPI(72.002845±3ppm)
79	8.09266	Cyclopentanone, 2-ethyl-	BPI(84.057094±3ppm)
80	8.09667	Acetoin	BPI(43.017989±3ppm)
81	8.15896	2-Octanone	BPI(58.041359±3ppm)
82	8.26667	Octanal	BPI(43.054356±3ppm)
83	8.38	cis-4-methoxy thujane	BPI(93.069790±3ppm)
84	8.39333	2-Propanone, 1-hydroxy-	BPI(43.017988±3ppm)
85	8.4	Hexanenitrile	BPI(54.033908±3ppm)
86	8.49	1-Hepten-3-one	BPI(55.017912±3ppm)
87	8.84924	Pyrazine, 2,5-dimethyl-	BPI(108.068186±3ppm)
88	8.51667	1-Octen-3-one	BPI(55.017982±3ppm)

89	8.52333	1-Ethyl-5-methylcyclopentene	BPI(81.069956±3ppm)
90	8.93333	2-Heptanol	BPI(45.033658±3ppm)
91	8.96667	2-Heptenal	BPI(57.033534±10ppm)
92	8.72333	2-Penten-1-ol	BPI(57.033509±3ppm)
93	8.99	Pyrazine, 2,6-dimethyl-	BPI(108.068171±3ppm)
94	9.1	Pyrazine, ethyl-	BPI(107.060384±3ppm)
95	9.2411	Cyclohexanone, 4-ethyl-	BPI(55.054280±3ppm)
96	9.25333	5-Hepten-2-one, 6-methyl-	BPI(43.017972±3ppm)
97	9.37	Pyrazine, 2,3-dimethyl-	BPI(108.068192±3ppm)
98	9.61	1-Hexanol	BPI(56.062071±3ppm)
99	9.66667	2-Hydroxy-3-pentanone	BPI(45.033623±3ppm)
100	9.96667	1-Hydroxy-2-butanone	BPI(57.033478±3ppm)
101	10.1433	2,5-Hexanedione	BPI(43.017994±3ppm)
102	10.19	Pyrazine, 2-ethyl-6-methyl-	BPI(121.076142±3ppm)
103	10.25	3-Hexen-1-ol	BPI(67.054244±3ppm)
104	10.2833	Cyclohexanone, 4-ethyl-	BPI(55.054235±3ppm)
105	10.31	Pyrazine, 2-ethyl-5-methyl-	BPI(121.076136±3ppm)
106	10.3733	2-Nonanone	BPI(58.041413±3ppm)
107	10.4967	Nonanal	BPI(41.038742±3ppm)
108	10.5767	Ethanol, 2-butoxy-	BPI(57.069914±3ppm)
109	10.6	Pyrazine, trimethyl-	BPI(122.083883±3ppm)
110	10.6333	2,4-Hexadienal	BPI(81.033597±3ppm)
111	10.7136	3-Octen-2-one	BPI(55.017905±3ppm)
112	10.7233	2-Hexen-1-ol	BPI(57.033626±3ppm)
113	10.8262	Cyclopentanecarboxaldehyde, 2-methyl-3-methylene-	BPI(67.054244±3ppm)
114	11.0333	2-Octanol	BPI(45.033615±3ppm)
115	11.19	2-Octenal, (E)-	BPI(41.038762±3ppm)
116	11.2208	Pyrazine, 2,6-diethyl-	BPI(135.091814±3ppm)
117	11.3167	Benzene, (2-methyl-1-propenyl)-	BPI(117.069975±3ppm)
118	11.3245	Thujon	BPI(81.069814±3ppm)
119	11.4433	Pyrazine, 3-ethyl-2,5-dimethyl-	BPI(135.091729±3ppm)
120	11.6167	Acetic acid	BPI(43.017982±3ppm)
121	11.6833	1-Octen-3-ol	BPI(57.033511±3ppm)
122	11.7582	1-heptanol	BPI(70.077768±3ppm)
123	11.7763	Pyrazine, 2,6-diethyl-	BPI(135.091739±3ppm)
124	11.87	Furfural	BPI(95.012793±3ppm)
125	11.9333	2,4-Heptadienal, (E,E)-	BPI(81.033576±3ppm)
126	11.96	2-Propanone, 1-(acetyloxy)-	BPI(43.017997±3ppm)
127	12.0111	5-Ethylcyclopent-1-enecarboxaldehyde	BPI(95.085581±3ppm)
128	12.3454	Pyrazine, 2-ethenyl-6-methyl-	BPI(120.068193±3ppm)
129	12.4667	1-Hexanol, 2-ethyl-	BPI(57.069929±3ppm)
130	12.7	Ethanone, 1-(2-furanyl)-	BPI(95.012704±3ppm)
131	12.8033	Oxalic acid	BPI(43.989386±3ppm)
132	12.8	cis-p-Mentha-2,8-dien-1-ol	BPI(119.085631±3ppm)

133	12.8767	3-Nonen-2-one	BPI(55.017878±3ppm)
134	12.9576	Hexanoic acid, pentyl ester	BPI(43.054365±3ppm)
135	13.0221	Benzaldehyde	BPI(105.033477±3ppm)
136	13.05	3,5-Octadien-2-one, (E,E)-	BPI(95.049184±3ppm)
137	13.0933	Furan, 2-methoxy-	BPI(83.012842±3ppm)
138	13.8267	1-Octanol	BPI(41.038736±3ppm)
139	13.23	6-Undecanone	BPI(43.054341±3ppm)
140	13.45	Propanoic acid	BPI(74.036291±3ppm)
141	13.6033	trans-2-Caren-4-ol	BPI(67.054252±3ppm)
142	13.8633	Longifolene	BPI(91.054286±3ppm)
143	13.99	Dimethyl Sulfoxide	BPI(62.989966±3ppm)
144	14.0733	2-Furancarboxaldehyde, 5-methyl-	BPI(110.036223±3ppm)
145	14.1267	Pyridine, 2-butyl-	BPI(93.057264±3ppm)
146	14.1867	2,3-Butanediol	BPI(45.033596±3ppm)
147	14.18	Isobornyl acetate	BPI(95.085484±3ppm)
148	14.4233	2,4-Octadienal, (E,E)-	BPI(81.033540±3ppm)
149	14.28	4-Cyclopentene-1,3-dione	BPI(96.020489±3ppm)
150	14.4711	Caryophyllene	BPI(91.054429±3ppm)
151	14.6546	Benzonitrile	BPI(103.041696±3ppm)
152	14.6333	Terpinen-4-ol	BPI(71.049258±3ppm)
153	14.7433	2(3H)-Furanone, dihydro-5-methyl-	BPI(85.028461±3ppm)
154	15.0833	Butyrolactone	BPI(42.046560±3ppm)
155	15.1933	Butanoic acid	BPI(60.020609±3ppm)
156	15.44	Benzoxazole	BPI(119.036612±3ppm)
157	15.4767	2-Decenal, (Z)-	BPI(55.054268±3ppm)
158	15.5333	Acetophenone	BPI(105.033538±3ppm)
159	15.7433	n-Caproic acid vinyl ester	BPI(43.054353±3ppm)
160	15.7667	2-Furanmethanol	BPI(98.036276±3ppm)
161	15.8239	1-Nonanol	BPI(55.054218±3ppm)
162	15.9583	2-Octenal, 2-butyl-	BPI(111.080232±3ppm)
163	16.05	2(5H)-Furanone, 5-methyl-	BPI(55.017933±3ppm)
164	16.3833	Methyl 1-methylpyrrole-2-carboxylate	BPI(108.044233±3ppm)
165	16.4	2,4-Octadiene	BPI(81.033544±3ppm)
166	16.4267	2(3H)-Furanone, 5-ethyldihydro-	BPI(85.028466±3ppm)
167	16.4667	2,4-Nonadienal, (E,E)-	BPI(81.033615±3ppm)
168	16.5233	Benzaldehyde, 3-ethyl-	BPI(134.072639±3ppm)
169	16.83	(-)-Carvone	BPI(82.041378±3ppm)
170	16.8671	Naphthalene	BPI(128.062083±3ppm)
171	16.9233	Pentanoic acid	BPI(60.020588±3ppm)
172	17.0433	2(5H)-Furanone	BPI(55.017911±3ppm)
173	17.1033	2(5H)-Furanone, 5-ethyl-	BPI(83.049164±3ppm)
174	17.2733	Oxime-, methoxy-phenyl-_	BPI(133.013579±3ppm)
175	17.3967	2H-Pyran-2-one, tetrahydro-6-methyl-	BPI(42.046530±3ppm)
176	17.5933	2,4-Decadienal, (E,E)-	BPI(81.033577±3ppm)
177	17.7416	Benzofuran, 2-methyl-	BPI(131.049209±3ppm)

178	17.8567	Hexanoic acid	BPI(73.02806±5ppm)
179	18.2667	Phenylethyl Alcohol	BPI(91.054203±3ppm)
180	18.3667	Benzene, 1-isocyano-3-methyl-	BPI(117.057335±3ppm)
181	18.5033	Heptanoic acid	BPI(60.020641±3ppm)
182	18.6047	Ethanone, 1-(1H-pyrrol-2-yl)-	BPI(94.028795±3ppm)
183	18.7667	Phenol	BPI(94.041332±3ppm)
184	18.8631	1H-Pyrrole-2-carboxaldehyde	BPI(95.036510±3ppm)
185	19.62	Phenol, 2-methyl-5-(1-methylethyl)-	BPI(135.080518±3ppm)

Table 3S. Selected volatile compunds identified using SPME-GCHRT-ToFMS on particular storage days. For comparison peak areas were used [TIC, 10^5 units]. The analyzed cold-pressed oils were obtained from gold (**GF**) and brown (**BF**) flaxseed, black (**BP**) and white (**WP**) poppy seed, white (**WS**) and black (**BS**) sesame, unroasted (**RA**) and roasted (**ROA**) argan, black cumin (**BC**), milk thistle (**MT**), raw pumpkin seeds (**RP**) and hemp (**HE**).

Compound	GF					BF					BP					WS					WP					RA					ROA					BC					MT					RP		HE		BS	
	0	2	4	7	10	0	2	4	7	10	0	2	4	7	10	0	2	4	7	10	0	2	4	7	10	0	2	4	7	10	0	2	4	7	10	0	2	4	7	10	0	2	4	7	10	0	10	0	10	0	10
Aldehydes																																																			
(E)-2-octenal	9.14	11.91	36.02	49.32	74.90		10.45	3.41	6.12	9.20	5.74	12.03	78.45	85.87	120.93						563.37	435.31	525.50	573.73	668.18	17.45	10.43	9.58	9.13	16.09											4.72	6.10	8.54	60.62	122.06	3.14	3.89	4.66	342.75		
(E,E)-2,4-decadienal																					59.81	23.86	56.25	77.21	568.73	3.10	9.78	6.50	4.71	2.31																		164.38		
(E,E)-2,4-heptadienal	2.05	21.68	264.04	607.48	1077.24	1.99	4.05	89.97	364.52	747.24			1.62	3.35	10.88						6.17	10.24	17.03	23.09	29.23																						3.37	631.75		
(E,E)-2,4-nonadienal											0.42		4.72	1.75	6.46						71.47	23.17	35.40	35.24	73.77																								22.94		
(E,E)-2,4-octadienal																					8.67	10.22	13.33	10.66	13.89																												
(Z)-2-decenal													5.55	1.57	6.75						22.91	12.05	14.37	14.19	23.14																												
2,4-hexadienal	1.24	3.56	14.80	25.10	51.96	40.72		12.60	22.78	40.72																																										
2-butenal		193.95	726.99	1373.45	1812.97	1457.03		490.96	1037.21	1457.03																																					105.03	1659.98		
2-butyl 2-octenal																					13.51	4.56	5.65	4.32	8.49																												
2-hexenal	4.50	4.98	11.11	22.76	41.87	1.27	1.57	1.09	14.46	17.72	1.07	4.21	12.61	22.41	43.88						28.15	62.56	86.14	122.92	138.56	3.32	1.89	2.33	2.38	4.46											2.15	3.13	5.52	16.73	36.45	2.70	3.07	3.14	99.24		
2-heptenal	7.09	8.90	48.28	76.36	120.87	8.82	5.30	34.30	56.83	90.91	4.35	11.31	58.35	105.57	233.02	4.61	0.90	1.04	2.83	9.34	170.68	299.01	410.01	515.56	560.42	12.02	10.92	11.04	14.78	28.53	5.39	7.99	7.51	8.34	15.23				2.47	2.13	5.13	13.91	28.91	87.79	156.30	14.85	60.39	1.62	30.56	2.91	8.64
2-methyl butanal																														1731.34	843.09	1658.03	1696.33	781.44						33.86	38.82	80.20	59.48	45.32			151.96	87.88		
2-methyl propanal																														490.62	154.48	416.25	578.50	128.65																
2-pentenal	9.86	62.91	460.67	892.38	1154.00	4.37	24.85	279.42	830.12	1301.19		2.72	11.01	28.32	52.66						13.92	295.80	311.74	390.24	442.06																	7.55	13.36	21.69	31.24			6.20	984.64		
2-propenal	2.84	114.91	731.67	1339.50	1451.99		23.21	307.03	757.66	950.68			111.38	152.12	340.18							308.73	361.30	393.89	359.71																	15.84	62.55	179.35	163.87			6.00	1084.29		
3-ethyl benzaldehyde	1.39	1.53	2.78	4.20	5.88		1.60	2.63	3.74	2.58			2.30	0.82	2.45						3.52	2.16	2.47	2.24	3.46	1.75	0.65	0.66	0.62	1.60	0.94	1.80		0.84	1.87						1.50	1.68	0.57	2.06	2.04			1.31	4.69		
3-methyl butanal																14.20	35.53	37.52	62.19	24.35						22.89	31.08	22.75	24.59	15.42											97.16	104.60	215.13	171.36	45.32	46.41	49.30				
5-ethylcyclopent-1-ene carboxyaldehyde																					9.50	10.08	10.57	11.31	10.91																												
benzaldehyde											5.87	3.75	9.41	6.33	10.27	7.46	3.68	3.00	3.94	7.75	92.09	70.59	77.00	62.74	68.36	6.12	3.08	2.53	2.67	4.55	12.21	11.49	12.10	11.59	16.84						18.25	18.20	17.63	18.02	23.54	7.11	11.39	27.59	84.35	5.37	3.85
butanal		8.12	18.53	35.63	48.49	89.63	6.58	12.60	57.90	89.63		22.03	19.99	102.12	31.41						74.38	206.56	192.90	172.81	181.55	11.06	8.60	8.82	10.54	11.82											7.33	10.36	37.78	67.76	36.70			8.00	98.97		
furfural											0.80	1.36	1.18	1.12							5.11	5.55	5.16	5.76	5.71						106.94	75.43	108.13	96.89	100.67						3.67	3.57	4.21	3.58	3.51	1.22	2.72		3.20	1.83	1.53
hexanal	23.21	54.56	160.19	181.95	195.54	35.51	42.84	127.35	190.28	196.84	324.17	576.41	458.27	1209.69	437.91	28.24	42.57	46.59	77.04	81.15	1046.95	2083.62	1944.18	1898.66	1832.15	211.41	226.81	226.85	270.91	216.68	118.49	133.20	190.72	195.05	184.77	0.57	1.20	1.41	2.17	3.46	55.65	107.78	253.75	491.58	554.47	75.45	234.47	44.38	897.69	13.72	79.37
heptanal	18.83	22.79	33.29	36.08	37.06	17.31	14.92	20.33	7.05	7.80	12.52	13.62	40.50	53.69	52.99						122.80	180.21	199.68	206.32	213.12	53.31	24.29	25.82	26.90	36.01	7.11	15.80	12.61	13.17	20.90						13.19	11.97	7.40	28.50	47.17	8.09	16.87	10.98	89.71		
nonanal	0.36	0.37	0.69	0.90	1.19	0.91	3.23	1.19	2.55	3.47	4.16	9.24	11.15	8.03	27.16						17.88	14.73	9.20	7.65	12.83	34.20	15.32	17.39	18.15	24.49	9.27	12.35	14.58	16.30	22.57						5.41	4.66	2.84	10.07	18.35			3.19	34.69		
octanal	4.70	6.14	12.63	16.71	23.81	5.48	6.78	2.02	4.29	5.19	5.85	5.44	19.87	24.29	26.85						38.84	35.07	33.76	38.25	43.66	25.19	9.37	9.95	9.85	16.08	3.71	8.27	4.88	5.58	10.58						5.87	5.54	1.96	15.13	29.12	5.30	6.06	4.40	23.42	1.21	1.70
pentanal	40.02	70.91	163.15	200.97	212.34	21.63	34.32	105.63	194.64	216.02	308.71	727.33	614.10	2335.08	823.13	41.45	35.12	36.88	60.38	79.67	1368.58	1576.39	2826.89	2609.82	2608.74	197.11	226.02	237.17	288.97	280.43	185.59	142.38	259.25	285.27	229.31						103.73	162.01	431.31	1345.82	1010.93	218.57	718.18	77.72	1541.42	20.56	97.84
propanal	8.43	250.36	735.85	1381.99	1218.07	33.40	261.75	1145.93	1886.51	2064.06	183.78		33.98	160.32	80.30								206.66	215.41	206.56	10.29	15.45	21.90	33.84	40.86											62.61	14.60	49.44	81.44	43.15			11.91	1158.65		
Ketones																																																			
2-butanone	158.02	172.73	157.01	244.49	271.18	535.65	525.47	503.19	687.05	759.24	65.61	52.26	64.73	143.85	73.80	62.09	51.88	57.62	82.43	99.94	787.87	2060.68	1810.76	1863.76	1799.55						315.19	135.27	321.25	342.32	159.33						279.74	160.67	144.18	7.96	98.67	166.23	453.30	140.44	554.49		
(E,E)-3,5-octadien-2-one	6.32	15.20	170.48	332.86	578.97	356.52		36.90	156.04	356.52											16.28	33.41	31.64	24.02	18.67																						3.47	231.47		
(R)-(+)-3-methyl cyclopentanone																					4.24	10.53	10.22	10.56	9.90																												
1- (acetyloxy)-2-propanone																														66.02	48.65	67.94	60.83	66.63																
1-hepten-3-one																					3.72	6.07	7.06	8.59	9.66																												
1-hepten-3-one																																								1.90	2.69	2.47	4.74	10.02						
1-hydroxy-2-butanone	1.77	41.50	153.71	184.23	237.30	346.45	25.29	149.80	252.06	346.45												8.97	22.15	24.53	25.99	26.41																								84.81		
1-hydroxy-2-propanone																														34.51	24.04	27.95	24.90	21.88																
1-octen-3-one																					47.70	92.77	108.21	119.98	116.33																				3.34	20.76				
1-penten-3-one	17.14	27.29	184.51	381.18	624.78		39.89	190.16	471.27	779.06																																										621.48		
2,3-butanedione																																															193.73	228.50				
2,5-hexanedione			17.69	34.13	45.92																																														
2-heptanone	10.88	12.04	24.95	43.94	60.15	16.37		5.41	15.03	16.37	49.55	48.79	88.68	283.14	244.85	8.04	4.60	4.06	3.69	6.02	1111.09	1936.17	1978.31	2149.40	2074.67	32.15	17.72	22.86	30.19	42.35	21.11	20.72	26.49	29.19	32.16						23.96	23.90	31.67	140.77	256.62	18.72	72.44	88.35	488.09		
2-hydroxy-3-pentanone	98.17	17.29	68.25	114.52	146.35			24.12	59.45	98.17																				12.06	13.61	13.92	11.48	16.28																
2-nonanone																					18.00	11.36	13.33	13.96	14.84																												
2-octanone											4.74	2.97	6.71	7.72	12.02						271.97	283.05	298.90	314.86	326.54																6.59	6.46	5.07	5.71	11.60	2.97	11.15	9.35	72.49		
2-pentanone								82.86	286.66	483.98											922.22	2146.76	2105.53	2132.22	2037.87	12.66	9.28	10.12	11.06	12.62											26.58	23.09	34.50	51.14	45.94			36.44	130.82		
3-hexen-2-one			5.20	20.51	36.59																																														
3-nonen-2-one																					43.21	30.58	41.91	38.87	63.17																												
3-octanone											6.33	15.03	4.06	7.02	9.93						134.71	225.17	239.75	258.40	231.11																									4.62	10.55		
3-octen-2-one											4.75	4.45	11.54	18.77	38.61						540.73	406.75	475.61	492.31	546.56	7.16	3.68	3.61	5.48	7.57	5.89	5.94	7.36	8.02	13.14						1.92	2.98	3.65	8.29	23.11						3.24
4-cyclopentene-1,3-dione																														3.80	3.04	4.20	3.83	3.70																
4-ethylcyclohexanone																					52.66	63.67	73.05	80.42	89.70																												
5-ethylcyclohexanone																					6.70	8.74	8.94	8.34	7.45																												
6-methyl-5-hepten-2-one																																															4.87	21.76				
6-undecanone																					32.80	12.50	14.48	11.53	16.61																												
acetophenone	4.29	3.16	5.78	5.22	7.45	4.19	1.85	2.36	3.53	6.83	4.60	2.20	2.33	1.51	2.23						4.27	4.51	4.39	4.19	4.44	1.57	1.04	0.86	1.35	1.30																						
Alcohols																																																			
1-hexanol	618.47	480.46	486.52	385.26	419.76	506.39	358.07	412.47	378.23	371.40	1199.94	958.60	890.48	924.25	884.59	162.18	117.36	102.87	124.71	149.71	1063.57	1632.68	1636.72	1585.99	1477.90	112.37	59.57	63.22	73.15	87.95	45.40	35.48	45.66	40.55	44.68	4.23	2.53	4.12	4.60	4.11	109.81	94.99	90.98	96.40	94.31	160.39	100.89	709.61	653.47	55.15	52.32
1-heptanol	13.00	9.59	15.24	13.88	18.11	2.97	2.27	3.74	5.23	6.74	9.69	7.34	13.12	17.69	17.89	4.30	1.79	3.91	2.25	4.31	37.50	39.85	43.04	40.90	41.13	13.93	6.68	7.59	8.84	11.70	5.69	5.79	6.17	5.66	7.01						3.81	3.38	2.20	5.46	10.03	3.73	4.12	6.83	7.68	1.76	1.17
1-metoxy-2-propanol																										17.66	9.19	8.52	8.77	11.14																					4.67	11.86
1-nonanol	2.86	2.38	3.49	3.16	4.39																																														
1-octanol	3.56	2.74	7.95	10.00	15.05	16.61																			3.60	4.58	3.00	2.94	5.36	2.52	3.07	3.28	2.28	3.94													2.73	24.21		
1-octen-3-ol	13.71	15.13	60.25	82.40	129.75	2.67	2.03	14.56	38.45	63.66	16.59	20.89	81.23	169.16	356.50						1761.31	1813.56	1975.41	2028.85	2149.76																11.87			92.90	193.56				690.89		
1-pentanol	152.62	141.12	130.28	120.65	125.38	124.38	93.43	101.21	113.94	129.21	514.83	434.23	332.80	614.96	436.50	55.05	54.57	48.97	61.68	64.26	1604.80	2962.76	2952.28	2963.63	2744.40	83.09	72.93	71.49	79.69	76.61	129.05	82.15	133.28	120.10	100.88						53.91	52.09	78.57	265.46	320.64	123.30	150.16	227.31	647.39	21.22	41.27
1-penten-3-ol	213.81	457.85	1397.85	2068.95	2148.01	191.78	288.28	1687.64	1739.04	2067.80											80.56	188.90	212.96	238.52	233.46																11.59	16.77	33.94	36.50	40.60			66.54	2312.56		
1-propanol	750.37	795.86	481.71	429.35	370.87	1123.71	959.51	870.35	910.44	902.15																																									
2,3-butanediol																														41.21	654.30	295.77	41.89	51.21																
2-butanol	25.00	26.89	15.21	12.84	11.11	385.15	365.51	323.79	294.20	248.52																																									
2-butoxyetanol																25.60	15.52	14.72	19.93	28.04																											109.00	131.91		
2-ethyl-1-hexanol																										6.01	3.38	3.89	4.40	5.90																					
2-hexanol											10.59	7.75	8.07	10.04	10.72						74.13	181.31	178.14	182.67	161.96																												
2-hexen-1-ol	22.67	19.34	22.32	18.25	22.84																																														
2-heptanol											20.20	14.66	17.46	21.78	24.12						256.86	382.92	366.18	367.51	345.02																									61.23	102.19		
2-methyl-1-propanol	229.53	249.15	181.14	164.79	146.17	252.97	229.99	236.93	234.14	224.73						23.22	20.42	25.41	22.25	30.28						79.36	107.72	143.87	161.83	68.63	386.82	143.77	344.60	348.07	150.74																	10.94	28.49
2-octanol											5.61	5.80	4.61	4.78	6.43																																				
2-pentanol	52.15	49.73	35.97	37.19	38.19	54.40	44.65	48.00	59.21	84.00	30.81	25.32	13.35	29.51	14.18	5.83	6.33	4.50	4.99	5.38	248.27	617.66	605.35	618.51	569.70																9.62	9.45	11.48	11.45	9.60	67.24	49.82	88.55	107.58		
2-penten-1-ol	23.35	21.73	45.11	77.04	111.98																																														
3-hexen-1-ol	15.04	12.01	13.04	12.95	13.67	7.09	4.64	6.80	7.13	9.05																																									
3-methyl-1-butanol	83.65	76.85	54.29	48.07	39.50	246.61	191.08	182.09	163.16	146.30	26.77	19.84	9.58	17.15	9.76	38.68	58.86	50.57	62.62	38.51						35.73	30.37	30.28	34.80	31.01	212.91	101.40	204.22	180.93	105.48						44.89	40.79	52.66	37.22	29.76	91.71	47.94	113.53	74.06	14.51	17.33
3-pentanol	59.62	57.36	38.91	36.07	33.34	119.66	96.89	96.11	99.43	107.23											17.32	37.38	40.68	44.03	41.13						33.90	16.18	31.82	29.53	16.73																
benzoxazole																					9.13	5.06	5.16	4.45	4.50																												
phenylethanol	6.09	3.83	6.93	3.96	6.71	3.03	2.34	3.17	2.99	3.81						3.47		1.46	1.74	2.93											1.14	2.35	1.60	1.81	3.10						4.43	3.70	2.17	1.87	4.12	2.15	1.46	4.93	9.41		
Acids																																																			
acetic acid	123.61	130.22	1160.62	2987.84	4165.05						64.29	113.73	249.36	424.71	659.48	270.38	71.15	165.02	205.12	333.15	492.26	672.68	842.05	1084.92	1200.24	334.87	137.00	177.09	191.28	418.52	351.16	377.71	604.04	557.37	692.01						643.41	696.11	869.35	1126.56	1225.96	362.10	494.18	785.67	3022.73	308.28	288.51
hexanoic acid			40.73	68.51	145.65	21.48		3.34	11.13	21.48	3.10	8.13	114.79	197.71	188.77						1778.63	1207.99	1927.90	1918.99	2037.26																4.09	4.21	8.12	38.52	268.59	5.82	8.42		678.58		
heptanoic acid																					91.27	66.01	79.71	73.52	121.29																												
butanoic Acid		2.00	35.13	79.16	133.60			7.21	27.04	49.74			10.49	18.62	22.73						78.41	91.41	111.61	119.80	137.45						9.71	29.43	23.52	19.13	38.87						4.66	7.29	6.13	8.97	25.96				382.13		
oxalic acid																																															323.96	477.56				
pentanoic acid									3.94	5.23		9.21	38.20	69.35	73.25						1142.40	1034.14	1336.89	1524.10	1747.47																		3.58	12.82	55.85	2.99	10.64		133.32		
propionic acid	6.60	19.33	270.66	776.42	1109.35	791.58		70.06	383.28	791.58																				14.82	16.76	28.86	15.55	24.95													10.93	789.32		
Heterocyclic compounds																																																			
butyrolactone	47.35	36.46	42.11	35.43	44.18	81.94	60.81	67.33	66.06	77.56	19.71	16.66	9.75	21.87	14.79	29.87	32.73	29.95	34.94	30.64	14.22	25.10	23.78	24.51	25.82	27.06	24.61	26.03	29.16	22.92	147.96	108.87	157.41	146.29	141.18						53.39	49.69	56.30	57.02	53.42	39.72	32.30	66.05	126.80	14.96	14.13
2-methyl-3-metylidenocyclopentane-1-carbaldehyde																					258.75	283.84	298.80	304.37	318.07																												
Furan derivatives																																																			
2-ethyl furan	360.11	438.84	456.27	700.96	899.06	117.83	122.89	245.21	815.45	1578.13	6.91	6.92	7.24	34.70	33.32	4.10	5.79	4.21	5.81	5.96	74.27	246.58	256.29	281.38	283.51																										105.30		
1- (2-furanyl)-etanone																														14.62	11.52	14.56	14.25	16.59																
2(5H)-furanone																														17.09	10.59	17.53	15.08	16.97						1.61	1.08	1.78	2.74	3.22	1.10	2.56				
2-furanmethanol																														14.43	16.58	18.37	15.75	23.40																
2-metoxy furan			11.20	14.82	20.31			3.67	8.53	13.34																																									
2-methylobenzofuran																																																			0.87	0.89
2-n-butyl furan	11.31	15.31	23.63	25.67	30.69						86.98	56.77	68.19	81.93	87.07	4.44	2.13	1.91	3.20	4.73	429.66	788.51	815.08	909.92	899.26	13.97	6.82	8.34	9.78	14.05													3.87	7.54	19.76	29.74						
2-propyl furan	7.22	7.32	1.35	8.99	10.27																122.79	108.22	118.14	138.95	122.20																												
5-etyl-2(5H)-furanone								11.77	28.26	50.30																														1.61	1.08	1.78	2.74	3.22						
5-etylodihydro-2(3H)-furanone	45.50	31.92	55.62	58.56	94.78	19.57	13.87	20.43	24.61	37.56	21.81	23.07	43.96	111.17	90.75						719.16	614.23	688.93	678.02	736.83	14.71	8.16	8.79	9.50	12.42	4.58	6.84	6.58	5.58	9.93						12.39	11.85	10.98	30.29	87.57	6.67	8.87	21.08	187.37		
5-methyl-2(5H)-furanone		0.90	5.91	12.55	23.35	23.21		4.87	13.10	23.21	21.81	23.07	43.96	111.17	90.75						1.91	2.18	2.12	1.88	2.85																								26.99		
5-methyl-2-furancarboxyaldehyde																														11.40	9.32	11.68	11.29	13.38																
dihydro-5-methyl-2(3H)-furanone	5.73	4.29	5.66	5.30	6.65						3.66	2.99	4.34	9.76	8.27	2.80	1.86	1.73	2.19	2.67	73.26	71.16	73.78	67.18	66.85	5.37	2.89	2.99	3.32	4.16											5.19	5.05	4.36	5.74	10.35			5.29	11.05		
Sulfur compounds																																																			
dimethyl disulfide																														1.59	3.55		6.40	3.86																
dimethyl sulfoxide						36.09	5.33	24.49	27.17	36.09						3.07	2.16	3.06	7.04	12.14	8.69	10.09	10.71	10.66	10.75																												
dimethyl sulfide						203.53	155.67																																												
Nitrogen compounds																																																			
1- (1H-pirrol-2-ill)-ethanone																														1.81	3.05	2.38	1.93	4.16																
1H-pirrol-2-carboxyaldehyde																														1.06	1.66	1.66	1.59	2.29																
1-izocyane-3-methylbenzene																2.45	1.04	0.91	0.99	2.28																																
1-methyl-1H-pirol																										135.17	87.93	81.54	85.99	88.18	1354.57	937.80	1364.20	1202.01	819.27																
1-methylpirrol-2-carboxylate methy																														2.98	3.37	3.89	3.49	4.94																
2,3-dimethyl pyrazine																														36.37	29.51	39.38	35.01	39.30																
2,6-diethyl pyrazine																														1.33	1.73	1.36	1.40	2.65																
2,6-dimethyl pyrazine																														195.22	172.37	215.39	204.14	223.52						17.31	16.95	19.84	13.96	17.38			10.75	10.19		
2-butyl pyridine																					7.25	4.70	4.56	3.16	5.79																												
2-ethenyl-6-methyl pyrazine																														2.07	2.61	0.35	2.20	3.63																
2-ethyl-5-methyl pyrazine																														97.54	91.25	109.44	102.15	127.15																
2-ethyl-6-methyl pyrazine																														25.76	34.48	29.44	28.03	45.97																
2-methyl-1-propenyl benzene	7.17	5.92	7.06	7.40	7.58																																														
3-ethyl-2,5-dimethyl pyrazine																														43.98	66.63	70.46	60.95	82.43																
benzonitrile	5.08	4.36	3.49	4.76	4.60	2.65	2.65	3.12	3.17	3.52																																									

2-methyl butanenitil															
3-methyl butanenitil															
ethyl pyrazine															
metoxyphenyl oxime	158.37	108.54	183.58	230.02	235.49	16.77	15.59	28.13	40.25	38.23	15.71	18.30	112.18	26.01	120.94
tetrahydro-6-methyl-2H-piran-2-on												1.04	2.15	4.25	3.53
trimethyl pyrazine															
Phenols															
phenol	6.51	6.18	7.63	7.82	8.24	3.39	2.43	3.78	5.39	6.29		2.20	4.33	3.34	4.73
2-methyl-5-(1-methylethyl)-pheno															
Terpenes															
trans-2-carene-4-o															
2,4-thujadiene															
3-carene	40.43	36.05	38.68	32.20	32.06						12.70	6.20	6.57	4.55	6.32
3-thujene	68.63	70.35	65.78	67.10	65.97										
α-phellandrene	3.52	2.59	2.36	4.45	2.69										
β-phellandrene	19.12	12.94	17.28	11.71	12.55										
camphen															
caryophyllene															
(-)-carvon											2.72	1.88	5.03	3.64	5.01
D-limonene	30.95	20.44	24.32	21.29	26.08	5.51	4.29	4.92	5.41		13.20	11.59	12.41	11.38	12.66
cis-p-menta-2,8-dien-1-o															
α-myrcene															
longifolene															
naphtalene															
α-ocimene	40.43	36.05	38.68	32.20	32.06										
α-pinene	68.63	70.35	65.78	67.10	65.97	156.99	115.98	128.97	118.09	108.54				20.58	42.81
β-pinene	11.27	10.23	10.51	10.27	11.35	12.47	9.33	11.11	10.06	12.05					
terpinen-4-o															
terpinolene															
β-tujone															

2-methyl butanenitil	19.05	30.71	29.18	36.00	20.66										
3-methyl butanenitil	23.75	38.40	34.52	40.51	25.41										
ethyl pyrazine															
metoxyphenyl oxime	73.85	15.36	16.64	15.44	58.50	75.11	41.33	51.55	58.22	58.24	73.03	20.56	17.07	18.22	54.28
tetrahydro-6-methyl-2H-piran-2-on						23.86	17.01	20.17	18.91	23.65					
trimethyl pyrazine															
Phenols															
phenol	9.19	4.29	4.21	4.61	8.71	5.46	6.20	7.73	7.74	8.60	5.70	3.24	3.57	3.76	4.84
2-methyl-5-(1-methylethyl)-pheno															
Terpenes															
trans-2-carene-4-o															
2,4-thujadiene															
3-carene	40.37	63.01	56.27	65.13	43.61										
3-thujene															
α-phellandrene	3.51	4.66	4.22	4.98	3.79										
β-phellandrene															
camphen															
caryophyllene															
(-)-carvon						22.74	8.12	11.92	9.29	15.40					
D-limonene	20.19	14.07	12.47	14.57	19.58	42.57	58.03	63.18	61.46	61.31	8.32	4.26	4.88	5.42	6.42
cis-p-menta-2,8-dien-1-o															
α-myrcene															
longifolene															
naphtalene	4.69	1.77	2.00	1.94	4.46										
α-ocimene															
α-pinene															
β-pinene	9.19	22.71	19.84	23.71	10.35										
terpinen-4-o															
terpinolene	1.74	1.25	1.22	1.36	1.49										
β-tujone															

2-methyl butanenitil															
3-methyl butanenitil															
ethyl pyrazine	53.76	49.51	57.54	53.11	66.41										
metoxyphenyl oxime	60.99	69.96	62.49	61.32	59.43						80.03	71.40	77.13	87.91	106.50
tetrahydro-6-methyl-2H-piran-2-on															
trimethyl pyrazine	92.83	106.40	122.90	110.89	138.78										
Phenols															
phenol	9.45	5.72	7.52	6.80	6.71						5.38	5.01	5.14	6.35	6.22
2-methyl-5-(1-methylethyl)-pheno						64.12	1.11	55.99	67.52	48.66					
Terpenes															
trans-2-carene-4-o						17.19	17.87	22.93	23.67	19.42					
2,4-thujadiene						16.79	13.88	16.87	17.26	15.87					
3-carene						5.00	5.48	5.82	5.83	4.96	185.05	172.30	244.75	129.24	120.07
3-thujene	46.59	32.36	42.72	40.34	40.90	2095.88	2139.43	2134.48	2168.21	2192.54					
α-phellandrene						437.38	449.71	454.51	459.56	457.23	75.53	73.06	38.90	19.38	48.94
β-phellandrene						13.69	13.88	15.36	36.05	13.59					
camphen						3.42	3.68	3.42	3.16	3.38					
caryophyllene															
(-)-carvon															
D-limonene	8.65	3.26	5.59	6.65	4.51	375.69	375.70	396.46	402.48	392.34	28.40	24.92	30.62	21.04	25.24
cis-p-menta-2,8-dien-1-o						31.38	25.64	30.09	30.60	26.17					
α-myrcene						17.37	16.25	21.36	21.28	17.64	12.45	12.91	13.49	7.35	9.43
longifolene						41.75	28.22	33.98	38.71	29.72					
naphtalene															
α-ocimene															
α-pinene						762.67	789.72	790.90	792.56	795.22	207.29	204.30	352.87	256.78	180.39
β-pinene	8.10	3.71	8.97	8.10	4.85	684.80	694.49	704.17	707.57	704.81	20.87	20.16	34.89	19.15	13.15
terpinen-4-o						46.04	33.84	41.68	43.81	36.31					
terpinolene						61.61	110.76	91.74	111.13	98.63	1.40	1.74	7.49	4.19	1.37
β-tujone						1.58	1.32	1.36	1.64	1.56					

2-methyl butanenitil			2.43	1.69		
3-methyl butanenitil						
ethyl pyrazine						
metoxyphenyl oxime	74.90	60.07	68.27	116.80	54.80	49.30
tetrahydro-6-methyl-2H-piran-2-on						
trimethyl pyrazine						
Phenols						
phenol	5.07	5.35		11.89	2.95	3.06
2-methyl-5-(1-methylethyl)-pheno						
Terpenes						
trans-2-carene-4-o						
2,4-thujadiene						
3-carene	179.20	171.50				
3-thujene	469.39	409.78	574.92	858.67		
α-phellandrene	5.10	3.90				
β-phellandrene			81.07	105.40		
camphen			3.75	9.25		
caryophyllene			1.37	5.56		
(-)-carvon			1.67	6.81		
D-limonene	38.07	39.00	46.17	70.41	9.32	6.99
cis-p-menta-2,8-dien-1-o						
α-myrcene						
longifolene						
naphtalene			2.90	5.21		
α-ocimene						
α-pinene	469.39	409.78				
β-pinene	71.09	62.63	190.25	245.67		
terpinen-4-o						
terpinolene						
β-tujone						
